# Transcription factor KLF6 upregulates expression of metalloprotease MMP14 and subsequent release of soluble endoglin during vascular injury

**DOI:** 10.1007/s10456-016-9495-8

**Published:** 2016-02-05

**Authors:** Eunate Gallardo-Vara, Francisco J. Blanco, Mercè Roqué, Scott L. Friedman, Toru Suzuki, Luisa M. Botella, Carmelo Bernabeu

**Affiliations:** Centro de Investigaciones Biológicas, CSIC, c/Ramiro de Maeztu 9, Madrid, 28040 Spain; Centro de Investigación Biomédica en Red de Enfermedades Raras (CIBERER), Madrid, 28040 Spain; Servei de Cardiologia, Hospital Clínic i Institut d’Investigacions Biomèdiques August Pi i Sunyer, Barcelona, Spain; Division of Liver Diseases, Icahn School of Medicine at Mount Sinai, New York, NY USA; Department of Cardiovascular Sciences, University of Leicester, Leicester, UK; National Institute for Health Research, Leicester Cardiovascular Biomedical Research Unit, Glenfield Hospital, Leicester, UK

**Keywords:** Endothelial cells, Vascular injury, Transcription, KLF6, MMP14, Soluble endoglin

## Abstract

After endothelial injury, the transcription factor Krüppel-like factor 6 (KLF6) translocates into the cell nucleus to regulate a variety of target genes involved in angiogenesis, vascular repair and remodeling, including components of the membrane transforming growth factor beta (TGF-β) receptor complex such as endoglin and activin receptor-like kinase 1. The membrane metalloproteinase 14 (MMP14 or MT1-MMP) targets endoglin to release soluble endoglin and is involved in vascular inflammation and endothelial tubulogenesis. However, little is known about the regulation of MMP14 expression during vascular wounding. In vitro denudation of monolayers of human endothelial cell monolayers leads to an increase in the KLF6 gene transcriptional rate, followed by an upregulation of MMP14 and release of soluble endoglin. Concomitant with this process, MMP14 co-localizes with endoglin in the sprouting endothelial cells surrounding the wound border. MMP14 expression at mRNA and protein levels is increased by ectopic KLF6 and downregulated by KLF6 suppression in cultured endothelial cells. Moreover, after wire-induced endothelial denudation, *Klf6*^+/−^ mice show lower levels of MMP14 in their vasculature compared with their wild-type siblings. Ectopic cellular expression of KLF6 results in an increased transcription rate of MMP14, and chromatin immunoprecipitation assays show that KLF6 interacts with MMP14 promoter in ECs, this interaction being enhanced during wound healing. Furthermore, KLF6 markedly increases the transcriptional activity of different reporter constructs of MMP14 gene promoter. These results suggest that KLF6 regulates MMP14 transcription and is a critical player of the gene expression network triggered during endothelial repair.

**Electronic supplementary material:**

The online version of this article (doi:10.1007/s10456-016-9495-8) contains supplementary material, which is available to authorized users.

## Introduction

The endothelium plays a crucial role in regulating pathophysiological processes like vascular injury, angiogenesis, vascular remodeling, tumor growth, vasoconstriction, vasodilatation, inflammation or blood vessel permeability. During all these processes, proliferation, migration and invasion of endothelial cells (ECs) are essential and the study of the regulatory mechanisms involved in endothelial gene expression is a necessary step in the search of therapeutic targets.

Upon vascular injury, a coordinated gene expression program is triggered among those genes coding for extracellular matrix proteins, growth factors, receptors and proteases. One of these classes of proteins is the transforming growth factor-β (TGF-β) family, which includes TGF-β, activins and bone morphogenetic proteins (BMPs). TGF-βs are angiogenic factors that target ECs during vascular wound healing and signal through the Smad family of transcription factors [1]. Mutations in endothelial components of the TGF-β system are responsible for several inherited vascular diseases [2]. Thus, heterozygous for mutations in the *ENG* (endoglin), *ACVRL1* (activin receptor-like kinase 1; ALK1), *GDF2* (BMP9) or *SMAD4* genes results in different variants of the familial vascular disorder known as hereditary hemorrhagic telangiectasia (HHT) characterized by an abnormal angiogenesis process where boundaries between arteries and veins are not well established [3, 4]. Endoglin protein is a membrane TGF-β co-receptor expressed primarily in ECs to regulate TGF-β signaling in the cardiovasculature [5, 6]. Endoglin is essential for angiogenesis during development, required for the maintenance of a normal vasculature in the adult, and mice that are homozygous for mutations in *Eng* die in mid-gestation from vascular defects [7–9].

The matrix metalloproteases (MMPs) represent an important family of proteins involved in diverse biological processes, including angiogenesis, stroke, vascular repair, inflammation and cancer [10–13]. Sustained MMP activity is associated with vascular pathologies such as hypertension, restenosis, vascular malformations and atherosclerosis [14, 15]. During angiogenesis, MMPs play a key role in extracellular matrix degradation, favouring endothelial cell migration and vascular invasion. Among these, MMP14 is a membrane-type MMP (MT-MMP) belonging to the collagenase type I class. In the vascular setting, MMP14 is involved in: (i) capillary tube formation of human ECs using 3D collagen matrices [10, 16]; (ii) the inflammatory response of ECs [17]; (iii) the atherosclerotic plaque vulnerability [18–20]; and (iv) the cross-talk between human airway basal cells and ECs [21]. Interestingly, MMP14 is upregulated in wound-derived blood vessels compared with vessels from intact human skin [22]. In addition to degrading the collagen surrounding new vascular cells [23], MMP14 releases latent TGF-β1 from latent TGF-binding protein (LTBP) and activates other MMPs secreted to the medium, such as MMP2, which has a critical role downstream of MMP14 for angiogenesis-mediated tumor invasion [24]. An increased number of MMP14 proteolytic substrates have been described recently, as well as its adhesive role in binding to collagens, glycoproteins, proteoglycans, integrins, cadherins and CD44 [17, 24]. In ECs, membrane endoglin is a proteolytic substrate of MMP14 resulting in the release of soluble endoglin [25, 26], which is associated with systemic hypertension during pregnancy and contributes to the pathogenesis of preeclampsia [26–29]. Soluble endoglin antagonizes the functions of its membrane-bound form by inhibiting angiogenesis, capillary tube formation and sprouting, and increasing vascular permeability [25, 27, 30]. However, little is known about the putative release of soluble endoglin during endothelial injury and how MMP14 gene expression is activated to cleave membrane endoglin.

The zinc finger Krüppel-like factor (KLF) family of transcription factors regulates diverse biological processes including proliferation, development, survival and responses to external stress [31, 32]. Several studies support an important role for this family of factors in vascular biology [33]. KLF6 is considered as a damage-response factor that promotes vascular remodeling because of its ability of transactivating several endothelial target genes by direct binding to their promoters [31]. Upon vascular injury, KLF6 expression increases and translocates into the nucleus to specifically enhance the transcriptional activity of endoglin and activin receptor-like kinase 1 (ALK1) [34, 35]; two key TGF-β receptors involved in vascular remodeling and angiogenesis [36]. KLF6 also targets a pool of genes involved in motility and invasion during vascular remodeling and angiogenesis including collagen α1 [37], E-cadherin, MMP9, tissue factor pathway inhibitor-2 (TFPI-2), urokinase plasminogen activator, IL6 and VEGF [31, 33, 34, 38]. Furthermore, KLF6 knock-out mice are embryonic lethal due to a failure of erythropoiesis and yolk sac vascularization [39]. Altogether, these data point to KLF6 as a key regulator of angiogenesis and vascular remodeling.

Here, we have addressed whether gene expression of MMP14 and its proteolytic activity on its substrate endoglin are deregulated upon vascular injury. We have also investigated the possible involvement of the transcription factor KLF6 during this process. Our results demonstrate that on vascular injury, KLF6 transcriptionally upregulates the expression of MMP14 that is associated with increased MMP14 proteolytic activity leading to the release of soluble endoglin from ECs.

## Materials and methods

### Cell culture

The primary culture of human umbilical vein-derived endothelial cells (HUVEC) was obtained from Lonza. These cells were cultured in early passages (3–10) and exponentially grown onto 0.2 % gelatin (Sigma) pre-coated plates in endothelial basic medium (EBM2) supplemented with endothelial growth medium (EGM2; Lonza). The human embryonic kidney (HEK293T) cell line was cultured in Dulbecco’s modified Eagle’s medium (DMEM; Gibco). Both cell types were cultured at 37 °C in 5 % CO_2_. All the media were supplemented with 10 % heat inactivated fetal bovine serum (FBS; Gibco), 2 mM l-glutamine, 100 U/mL penicillin and 100 µg/mL streptomycin (Gibco).

### Endothelial wound healing assay

For wound healing assays, cells were grown to confluence onto 0.2 % gelatin pre-coated wells. The wound was created by scratching the cell monolayer using a sterile pipette tip. Then, plates were immediately washed twice with PBS to remove the remaining cells and fresh medium was added. Endothelial cell migration into the denuded area was monitored with photographs taken at the time of the wound (0 h) and 2, 4, 6, 8 and 24-h post-wound. The Image J program was used to quantify the wound healing process by measuring the distance between the edges of the denuded area.

### Mice and mechanical injury experiments

Generation of *Klf6*^+/−^ mice in the C57BL/6 strain has been previously reported [39]. To monitor the expression pattern of MMP14 in a vascular injury, we followed the technique described by Garrido et al. [34]. Briefly, *Klf6*^+/+^ and *Klf6*^+/−^ mice were anesthetized and underwent bilateral endoluminal injury to the common femoral artery by passing three times a 0.25-mm-diameter angioplasty guidewire as described [40]. At 28 days post-injury, mice were killed and approximately 2-mm-thick transverse segments were cut at the level of the injury, in previously isolated hind limbs blocks embedded in paraffin. Five-µm cross-sections were obtained throughout the injured fragment.

### Inmunohistochemistry

Paraffin embedded sections of femoral arteries were prewarmed at 60 °C and deparaffinized with xylene prior to hydration with a series of ethanol-graded dilutions followed by distilled water. Slides were subjected to antigen retrieval with 0.01 M sodium citrate buffer, pH 6.0 for 45 min at 95 °C in a water bath. The endogenous peroxidase activity of the tissues and unspecific epitopes was blocked with “peroxidase blocking reagent” during 5 min and “protein blocking reagent” during 30 min, respectively. All the reagents used were from NovoLink Polymer Detection System Kit for IHC (Novocastra, Millipore). MMP14 staining was detected with a rabbit monoclonal anti-MMP14 antibody (ab51074, Abcam) incubated overnight at 4 °C. Then, samples were incubated with the secondary antibody biotin-goat anti-rabbit IgG (H + L), followed by incubation with streptavidin–HRP (Cat #21126; Pierce). For development of the peroxidase activity, 3,3′-diaminobenzidine (DAB) chromogen was used. Nuclei were counterstained with Mayer’s hematoxylin 0.02 %, followed by immersion in ammonia water. Finally, slides were mounted in HiMo (05-HM, Bio-Optica, Milano, Italy) for observation with a camera-coupled bright-field microscope.

### ELISA of soluble endoglin and IL-6

HUVEC monolayers were scratched with a pipette tip as described above. Culture supernatants were collected at the indicated times and analyzed by ELISA assays. Concentrations of human soluble endoglin and human IL-6 in the cell culture media were determined according to the manufacturer protocol by Quantikine Human Endoglin/CD105 and Quantikine Human IL-6, respectively (DNDG00 and D6050; R&D Systems). Soluble endoglin was also measured in the culture media of HUVECs or HEK293T cells previously transfected with expression vectors, as indicated. All immunoassays were measured in a GloMax multidetection system (Promega) and normalized by the percentage of cells in each control and post-wounded well.

### Inmunofluorescence microscopy

In order to monitor the co-localization of MMP14 and endoglin in the membrane during the wound healing process, HUVECs were grown to confluence onto 12-mm-diameter coverslips, previously coated with 0.2 % (v/v) gelatin (Sigma-Aldrich) in PBS. When necessary, HUVECs were nucleofected with KLF6 plasmids and siRNAs for 48 h, prior to the wound healing assay. Then, the endothelial monolayer was disrupted once with a micropipette tip. After 4 and 6 h, cells were fixed with 3 % paraformaldehyde in PBS and blocked with PBS-BSA 1 %, prior to the incubation with the primary antibody mouse antihuman endoglin (P4A4; DHSB). Afterward, samples were washed with PBS twice and incubated with the second primary antibody, monoclonal rabbit antihuman MMP14 (ab51074, Abcam). Then, cells were incubated with two different secondary antibodies: Alexa 488 goat anti-mouse IgG and/or Alexa 647 goat anti-rabbit IgG (Molecular Probes, Invitrogen). To visualize the samples and for nuclear staining, the slides were mounted in Prolong-DAPI reagent (Molecular Probes, Invitrogen). Samples were observed using a fluorescence confocal microscopy Sp5 (DMI6000 CS Leica Microsystems). For the semi-quantification of fluorescence intensity and co-localization of endoglin and MMP14, an analysis of scatter plots (cytofluorograms) was carried out in the central layers of each condition. Data about the distribution of fluorescence signals and specific overlapping pixels were obtained taking into account Pearson’s correlation and setting a common background and threshold for green and red channels. All these measurements were obtained using the Image J and LAS-AF 2.6 software.

### In silico analysis of promoter sequences

*In silico* analysis of GC-boxes containing putative KLF6 and Sp1 motifs in the MMP14 proximal promoter, corresponding to the upstream sequences of human (ID: 4323) and mouse (ID: 17387) genes, was performed by the Genomatix MatInspector software tool (http://www.genomatix.de/products/MatInspector).

### Flow cytometry

HUVECs were collected at different times (0, 2, 4, 6 and 8 h) after injury for the analysis of MMP14 and endoglin expression levels at the endothelial cell surface. Cells were incubated with a mouse monoclonal antibody against human endoglin (CD105; P4A4) or a rabbit monoclonal antibody against human MMP14 (ab51074, Abcam). A negative control was included using an irrelevant isotype control antibody X63 (IgG1). After washing the cells, they were incubated with Alexa Fluor 488 goat anti-mouse (A-11001 Molecular Probes, Invitrogen) for endoglin detection or Alexa Fluor 488 goat anti-rabbit IgG (11008 Molecular Probes, Invitrogen) for MMP14 detection. When working with nucleofected HUVECs, the expression levels of endoglin and MMP14 were measured by incubation with directly conjugated primary antibodies: Alexa Fluor 488 antihuman MMP14 (#128527; R&D Systems) and APC anti-endoglin/CD105 (#166707; R&D Systems). The fluorescence intensity was estimated with an EPICS XL and FC 500 flow cytometers (Beckman Coulter). A minimum of 10,000 cells were counted for each experimental point.

### Metalloproteinase activity

Metalloproteinase activity of HUVECs was determined using the fluorogenic peptide substrate Mca-PLGL-Dpa-AR-NH_2_ (ES001; R&D Systems). HUVEC monolayers, at control conditions and at different times after wounding, were washed and incubated with 3 µM fluorogenic peptide diluted in DMSO and TNC buffer (50 mM Tris, 0.15 M NaCl, 10 mM CaCl_2_ and 0.002 % NaN_3_; pH, 7.5) during 1 h at 37 °C. The reaction was stopped by adding stop solution 10X (100 nM EDTA + 0.02 % NaN_3_). The fluorescence signal was read with a Varioskan flash spectral scanning multimode reader (Thermo Scientific).

### Quantitative real-time PCR

Total mRNA was isolated with the SpeedTools Total RNA Extraction Kit (Biotools). A total of 1 µg of RNA was retrotranscribed with a high capacity cDNA reverse transcription kit (170-8891; iScript cDNA Synthesis kit; BioRad). The resulting cDNA was used as a template for quantitative real-time PCR (qRT-PCR) performed using LightCycler 480 PCR Master SYBR Green (Roche Applied Biosciences). Oligonucleotides for selected genes were designed using Roche software for qRT-PCR. The following forward (Fw) and reverse (Rv) primers were used: hENG, Fw 5′-GCCCCGAGAGGTGCTTCT-3′ and Rv 5′-TGCAGGAAGACACTGCTGTTTAC-3′; hKLF6, Fw 5′-CGGACGCACACAGGAGAAAA-3′ and Rv 5′-CGGTGTGCTTTCGGAAGTG-3′; hIL6, Fw 5′-GAAGGCAGCAGGCAACAC-3′ and Rv 5′-CAGGAGCCCAGCTATGAACT-3′; hMMP14, Fw 5′-CGATGTGGTGTTCCAGACAA-3′ and Rv 5′-TGGATGCAGAAAGTGATTTC-3′. As an internal control, mRNA levels of 18S were measured using primers Fw 5′-CTCAACACGGGAAACCTCAC-3′ and Rv 5′-CGCTCCACCAACTAAGAACG-3′. Amplicons were detected using a LightCycler 480 System II-384 (Roche Applied Biosciences). The specificity of the PCR products was assessed by melting curve analyses which showed only single amplified products. Assays were done in triplicate, and the results were normalized according to the expression levels of 18S rRNA.

### Cell transfection, plasmids and reporter assays

Transient HEK293T transfections and HUVECs nucleofections were carried out using Lipofectamine 2000 (Invitrogen) and Amaxa HUVEC Nucleofector kit (VPB-1002, Lonza), respectively, according to the manufacturer’s instructions. Cells were analyzed 48 h later for protein and mRNA expression by flow cytometry or qRT-PCR. Human KLF6 was overexpressed by the encoding vector pCiNeo/KLF6 [35]. The siRNA-mediated KLF6 knock-down was carried out using a pool of five siRNAs targeting human KLF6 (sc-38021; Santa Cruz Biotechnology) and scrambled siRNA (Universal negative control SIC001; Sigma), as a negative control. The pMax-GFP vector (Amaxa Biosystems) was used as a control to assess transfection efficiency. For MMP14-luciferase reporter assays, three different 5′-serial constructs (−1544/+235; −800/+235; −300/+235) of the MMP14 promoter (pMT1) were chemically synthesized and cloned (BioNova Cientifica SL) into the pGL2-luc basic reporter plasmid (Promega). HEK293T cells were co-transfected with 1 µg/well of pGL2-luc basic promoter, pGL2-luc-pMT1-1544, pGL2-luc-pMT1-800 or pGL2-luc-pMT1-300 and Mock or pCIneo-KLF6 expression vector [34] for each condition. Forty-eight hours after transfection, cell lysates were analyzed using dual-luciferase reporter assay system (Promega) in a GloMax multidetection system luminometer (Promega). Transfection efficiency was normalized to β-galactosidase luciferase activity.

### Chromatin inmunoprecipitation assays (ChIP)

ChIP experiments were performed with ChIP-IT Express Enzymatic kit (#53009; Active Motif) in HUVECs, as described by Garrido et al. [34]. Briefly, aliquots of the final sheared chromatin were used as “input chromatin” and the rest was incubated with protein G magnetic beads and 10 μg of rabbit polyclonal antibody antihuman KLF6 (#R-173; sc-7158, Santa Cruz Biotechnology), rabbit polyclonal antibody anti-histone H3K4me2 (#39141; Active Motif) or control rabbit IgG (#sc-2027; Santa Cruz Biotechnology). Protein G magnetic beads bound to the immune complexes were pelleted, washed and eluted. Then, cross-linking was reversed and samples were incubated with proteinase K and precipitated prior to resuspension in TE buffer. Binding of KLF6 was analyzed by PCR using primer pairs of four main KLF6-sites rich regions in the MMP14 promoter sequence. The first region encompasses from −1142 to −857 (285-bp), the second one from −783 to −569 (214-bp), the third one from −107 to +72 (179-bp) and the fourth one from −7 to + 233 (240 bp). Sequences of primer pairs were: first region, Fwd 5′-AGACTTCTATTCTTCTGCCA-3′ and Rev 5′-ATAAAGCTGACCGTGAGA-3′; second region, Fwd 5′-CTTTAAGAATTGCCTCCTTT-3′ and Rev 5′-CCAACCTTTGTAGAAAGACA-3′; third region, Fwd 5′-GGCTAAAACAACCACGTC-3′ and Rev 5′-GTGCCTGTTTGCTCTTCT-3′; and fourth region, Fwd 5′-AGGGAACCAGACCCCAGTTCG-3′ and Rev 5′-TCCGAGACCACCGGGTCA-3′. For negative and positive control PCRs, primers from ChIP-IT control human kit (#53010; Active Motif) were used.

### Statistics

Data were subjected to statistical analysis, and the results are shown as mean ± SD. Differences in mean values were analyzed using Student’s *t* test. In figures, statistically significant values are marked with asterisks (*p < 0.05; **p < 0.01; ***p < 0.005; *ns* not significant).

## Results

### MMP14 and endoglin protein expression increases during endothelial denudation

**Fig. 1:**
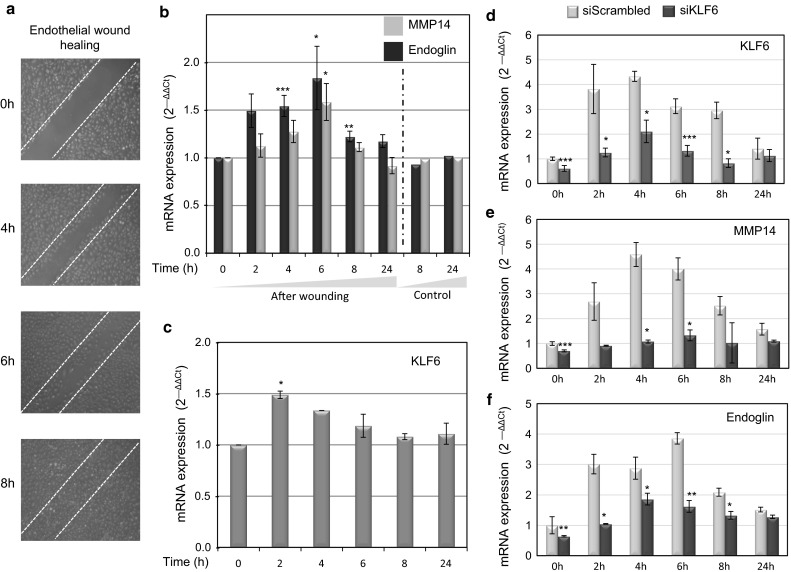
MMP14 and endoglin mRNA levels increase in endothelial cells after in vitro denudation. **a** HUVECs were wounded in vitro, leaving approximately 40 % intact of total monolayer. Photographs were taken at different times after wounding (0–8 h). Cells were lysed at the times indicated (0–24 h), and RNA was extracted and processed for qRT-PCR of human MMP14 and endoglin genes (**b**). KLF6, as a primary response gene to injury, was also quantified (**c**). mRNA expression levels of KLF6 (**d**), MMP14 (**e**) and endoglin (**f**) genes were measured upon KLF6 suppression with siRNA specific for KLF6 (siKLF6), using scrambled siRNA as a control. Fold change of mRNA expression levels with respect to basal conditions are indicated. Results were normalized to gene expression levels of 18S rRNA, as a housekeeping gene that does not change during the wound healing process. *p < 0.05; **p < 0.01; ***p < 0.001 with respect to basal conditions

**Fig. 2:**
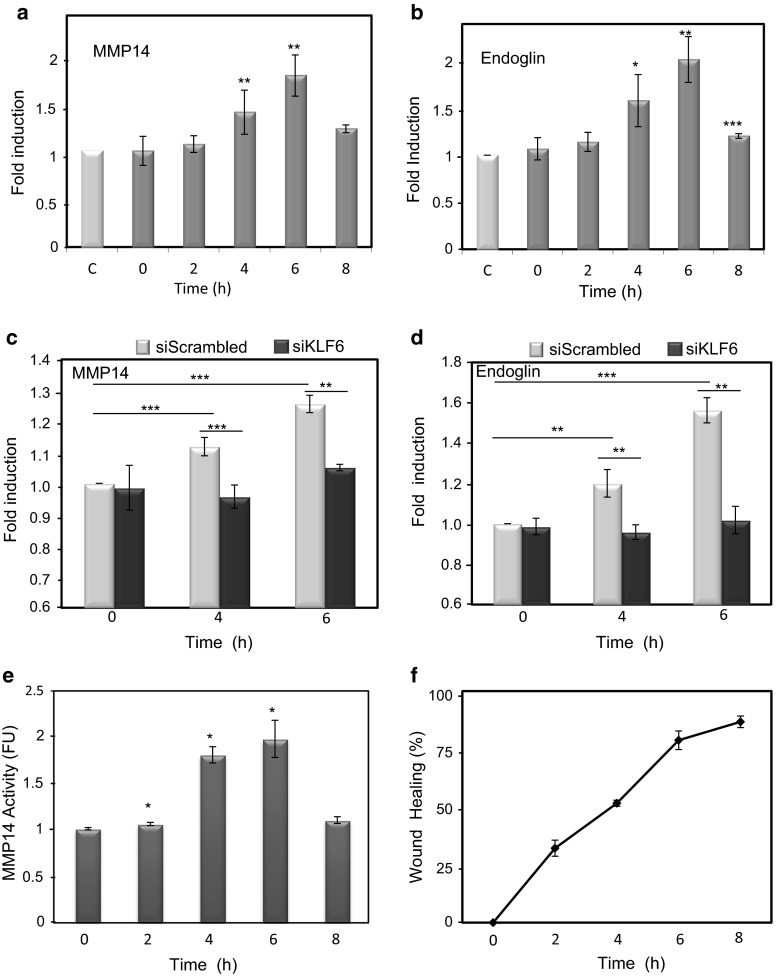
MMP14 protein expression and activity are increased during wound healing. MMP14 (**a**) and endoglin (**b**) protein levels on the surface of HUVECs at different time points during wound healing experiments were analyzed by flow cytometry as described in “Materials and methods.” Histograms indicate protein levels represented as fold induction with respect to resting cells at 8 h (control, C). MMP14 (**c**) and endoglin (**d**) protein levels on the surface of HUVECs at different time points after wound healing were measured by flow cytometry upon KLF6 suppression with siRNA specific for KLF6 (siKLF6). Scrambled siRNA (siScrambled) was used as a control. Histograms indicate protein levels represented as fold induction with respect to cells at 0 h. **e** MMP14 activity was measured during endothelial wound healing using a fluorogenic peptide and represented as a fold induction with respect to cells at time 0 h. **f** The percentage of endothelial healing is represented at different time points after wounding. Cells at approximately 80 % confluency (6 h after wounding) showed the highest MMP14 activity. *p < 0.05; **p < 0.01; ***p < 0.001 with respect to control condition (**a**, **b**), to time 0 h (**e**), or as indicated (**c**, **d**)

We have previously demonstrated that following endothelial injury, KLF6 expression increases and translocates into the nucleus to specifically enhance the transcriptional activity of membrane-bound endoglin [35]. Because MMP14 can target membrane endoglin to release soluble endoglin [25, 26], we wondered whether MMP-14 expression was also modulated during endothelial wound healing. After denudation injury experiments performed in human umbilical vein endothelial cells (HUVECs) (Fig. 1a), MMP14 and endoglin expression was upregulated at mRNA (Fig. 1b) and cell surface protein (Fig. 2a, b) levels during the central hours (4–6 h) of the healing process. As expected, KLF6, a primary response gene to injury, was upregulated at early stages (2 h; Fig. 1c) of the wound healing process, an interval that is compatible with its transcriptional involvement in the subsequent increased expression of endoglin [35]. Interestingly, suppression of KLF6 in HUVECs (Fig. 1d) markedly inhibited the injury-induced expression of MMP14 and endoglin at mRNA (Fig. 1e, f) and protein (Fig. 2c, d) levels. The upregulation of MMP14 suggests an active role for its catalytic activity during the wound healing process. Indeed, using a fluorogenic peptide as a substrate, the proteolytic activity of MMP14 was steadily increased during the endothelial repair process (Fig. 2e, f). Among others, the proteolytic activity of MMP14 may contribute to degrade extracellular matrix proteins, as a typical collagenase that, in turn, could facilitate the associated endothelial cell migration during the wound healing process [25, 41, 42]. Accordingly, we found that suppression of MMP14 leads to a significantly decreased rate of wound closure in HUVEC monolayers upon denudation (Supplementary Fig. 1a–d).

### Endothelial injury induces co-localization of MMP14 and endoglin and the release of soluble endoglin

**Fig. 3:**
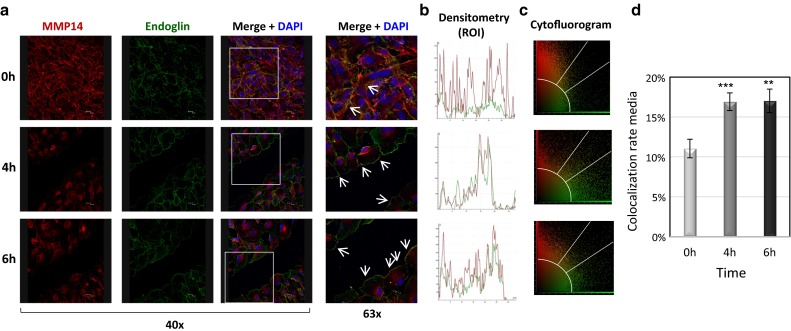
MMP14 and endoglin co-localize upon endothelial denudation. HUVEC monolayers were wounded in vitro, and the presence of MMP14 and endoglin was detected by immunofluorescence at the indicated time points. Cells were incubated with a mouse antibody anti-endoglin (P4A4) or a rabbit antibody anti-MMP14, followed by a secondary anti-mouse IgG coupled to Alexa 488 (*green staining*) or a secondary anti-rabbit IgG coupled to Alexa 647 (red staining). (**a**) Single staining and merge images plus DAPI (nuclear staining in *blue*) are shown at the indicated magnifications (40× and 63×). The *arrows* indicate sites where MMP14 and endoglin co-localize (*yellow color*). A magnification (63×) of the merge images (area within the *square*) is shown in the right column. (**b)** Using merge images (63×), measurements of endoglin (*green*) and MMP14 (*red*) along 30-µm length longitudinal sections of representative membrane areas (indicated by *arrows*) of each condition were carried out. Fluorescence intensities were measured and represented in histograms using Image J software tool. Densitometry profiles of the region of interest (ROI) and distributions of both signals at distinct time points (0, 4, and 6 h) are shown. **c** Representative merge images showing the co-localization of MMP14 and endoglin (area within the *two white lines*) in cytofluorograms. This scatter plot shows an increased co-localization area/area foreground (or total fluorescence area) upon endothelial wounding. **d** Quantification (percentage) of MMP14 and endoglin co-localization obtained from the cytofluorograms. **p < 0.01; ***p < 0.001 with respect to time 0 h. This is a representative experiment of ten different ones

The parallel increase in MMP14 and endoglin protein levels, as well as the enhanced MMP14 proteolytic activity observed during the wound healing process, prompted us to test whether MMP14 could target membrane endoglin to release its soluble form. Supporting this hypothesis, co-localization of MMP14 and endoglin in the plasma membrane of HUVECs under basal conditions and after 4 and 6 h of denudation was observed by confocal microscopy (Fig. 3a). In agreement with Fig. 2, an increased expression of both proteins was detected at 4–6 h after wounding, a time period associated with an active cellular migration. Immunostaining of MMP14 was observed in the membrane of those cells close to the edge of the wound. Some of these cells showed a clear co-localization of MMP14 and endoglin, as depicted in the merged images (yellow staining). Control staining in the absence of primary antibodies did not show any positive signal (data not shown), excluding a potential trapping of the immunofluorescent secondary antibodies, especially by the cell monolayer in the front of advance. In addition, a parallel distribution of MMP14 and endoglin was found along a drawing line above the edge of the wound after 4 and 6 h of injury, whereas an irregular distribution was found in the resting condition of the confluent HUVEC monolayer (Fig. 3b). A scatter plot (cytofluorogram) analysis of both fluorescent signals also showed an increased co-localization area/area foreground (or total fluorescence area) at 4 h and 6 h after wounding with respect to time 0 (Fig. 3c, d). Moreover, suppression of KLF6 with siRNA led to a decrease in the MMP14 and endoglin co-localization rate after wounding associated with a marked decrease in their basal expression (Supplementary Fig. 2). Further support for the association between endoglin and MMP14 was provided by co-immunoprecipitation studies (Supplementary Fig. 3). Thus, endoglin was detected by Western blot analysis after immunoprecipitation with anti-MMP14 antibodies in HUVECs, and this endoglin signal was enhanced at 6 h after wounding or upon ectopic expression of KLF6 (Supplementary Fig. 3a). By contrast, the intensity of the MMP14-associated endoglin band was decreased upon silencing of KLF6 with siRNA (Supplementary Fig. 3b).

**Fig. 4:**
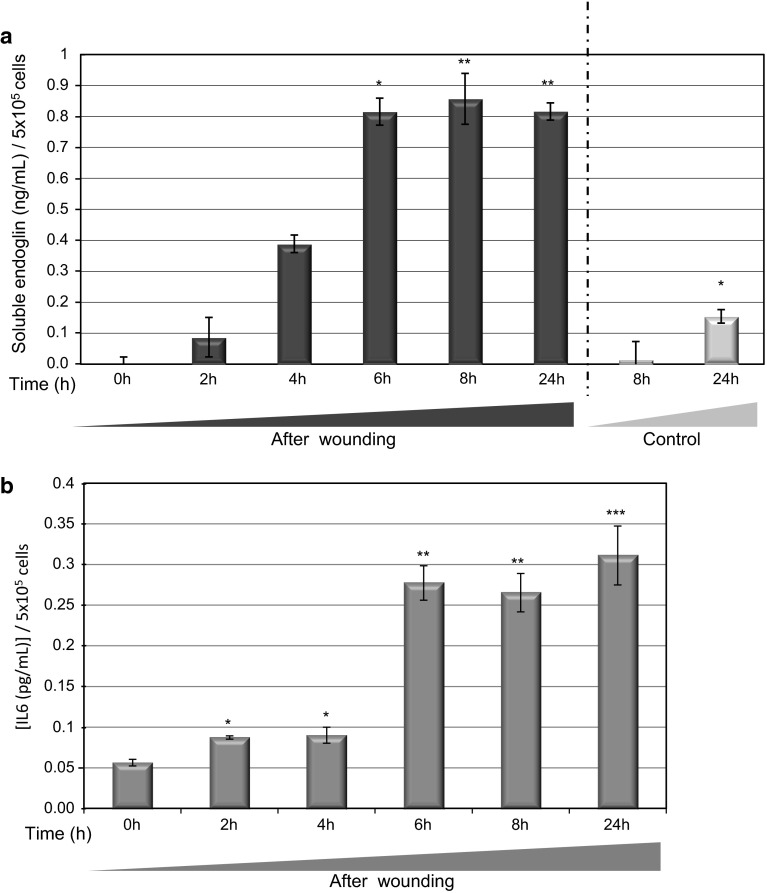
Soluble endoglin is released during wound healing of endothelial cells. HUVEC monolayers were wounded in vitro, culture supernatants were collected at different times, and levels of soluble endoglin (**a**) and IL6 (**b**) were measured by ELISA and compared to unwounded cells (control). Concentrations of soluble endoglin and IL6 are referred to 5 × 10^6^ cells. Basal levels of unwounded cells were substracted from IL6 levels of cells after wounding at the same time points. *p < 0.05; **p < 0.01; ***p < 0.001 with respect to time 0 h condition

Next, we assessed whether the co-localization of MMP14 and endoglin was associated with an increased shedding of soluble endoglin. Indeed, levels of soluble endoglin were markedly increased in the culture medium of HUVEC monolayers at different time points after denudation, showing the maximum endoglin shedding at 8 h after wounding (Fig. 4a). Furthermore, suppression of MMP14 in HUVEC monolayers led to significantly decreased levels of soluble endoglin at 6 and 8 h after wounding compared to controls (Supplementary Fig. 1e, f). In agreement with a recent report [34], IL6 levels were also induced upon endothelial denudation, showing a similar kinetics as soluble endoglin (Fig. 4b).

### KLF6 upregulates expression of MMP14 after endothelial injury

**Fig. 5:**
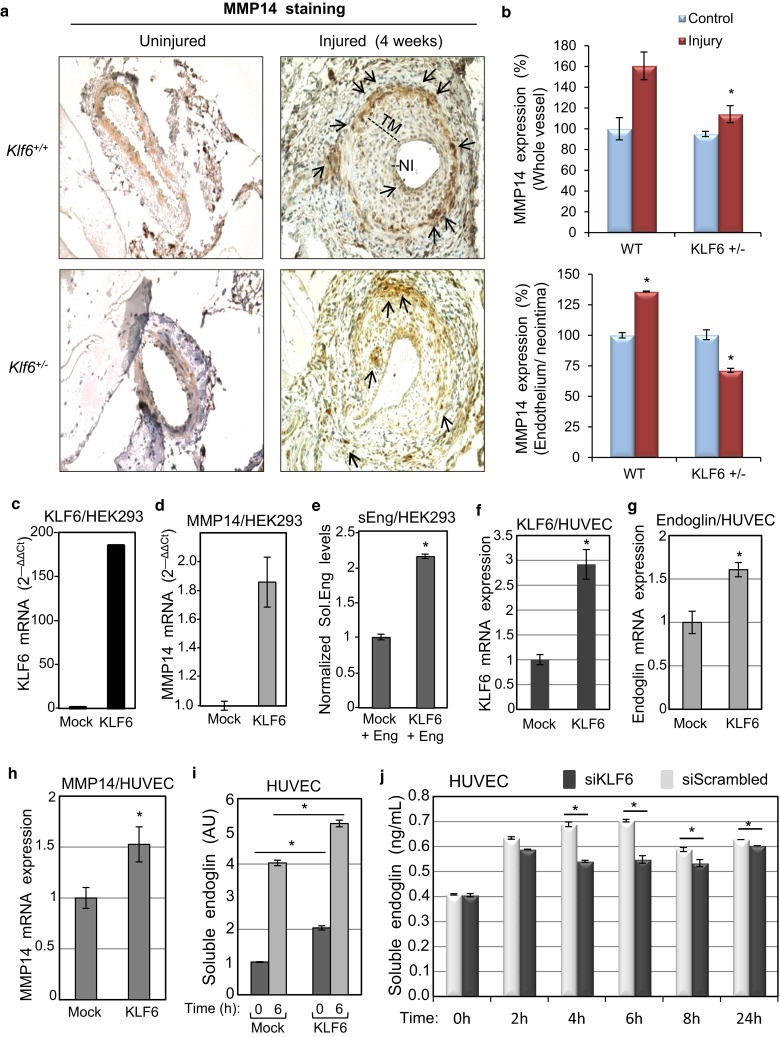
KLF6 regulates MMP14 expression. *Klf6* ^+*/*−^ heterozygous mice express lower levels of MMP14 after endothelial injury. **a** Immunohistochemical staining of MMP14 in mouse femoral artery after endothelial injury (4 weeks) compared to uninjured samples from *Klf6* ^+/−^ heterozygous and *Klf6* ^+^/+WT mice. Pictures were taken at ×25 magnification. In WT animals, MMP14 expression is upregulated (*black arrows*) in neointima and tunica media of femoral arteries after endothelial injury and this upregulation was less pronounced in *Klf6* heterozygous mice. **b** Histograms representing the MMP14 media expression samples (*n* = 5 for each condition) of femoral arteries after endothelial injury comparing WT and *Klf6* heterozygous mice. Measurements of whole vessels (*upper histogram*) and endothelium/neointima (*bottom histogram*) were normalized taking into account the width of vessels or neointima, respectively (*p < 0.05 with respect to control condition). NI, neointima; TM, tunica media. **c**–**e** Overexpresssion of KLF6 increases MMP14 mRNA expression and soluble endoglin release in cultured cells. HEK293T cells were transfected with expression vectors encoding KLF6 (pciNeo KLF6) or endoglin (Eng) and an empty vector (Mock), as indicated. Quantification by qRT-PCR of ectopic KLF6 (**c**) and endogenous MMP14 (**d**) transcript levels. Levels of soluble endoglin released to the medium were measured by ELISA (**e**). **f**–**i** Overexpresssion of KLF6 increases MMP14 mRNA expression and soluble endoglin release in HUVECs. HUVECs were nucleofected with an expression vector encoding KLF6 (pciNeo KLF6) or an empty vector (Mock), as indicated. Quantification by qRT-PCR of KLF6 (**f**) and endogenous endoglin (**g**) and MMP14 (**h**) transcript levels. Levels of soluble endoglin released to the medium from HUVECs subjected to endothelial denudation for 6 h were measured by ELISA (**i**). **j** KLF6 suppression was carried out in HUVECs by nucleofection with siRNA specific for KLF6 (siKLF6), using Scrambled siRNA (siScrambled) as a control. Nucleofected HUVECs were wounded in vitro and supernatants were collected at different times. Soluble endoglin levels were measured by ELISA, as described in Materials and Methods. In **c**–**i**, values were normalized to mock or untreated cells at time 0 h. *p < 0.05 as indicated

KLF6 is a common transcription factor that is upregulated at the beginning of the activation phase in the endothelial injury model, as shown in Fig. 1c, in agreement with previous reports [34, 35]. This kinetics induction of KLF6 is compatible with the subsequent increase in MMP14 protein levels. To assess the effect of Klf6 suppression in vivo, MMP14 expression was studied in heterozygous *Klf6*^+/−^ and wild-type mice, using a model of wire-induced endothelial injury. Four weeks after removal of the tunica intima (endothelial layer) from the hindlimb femoral artery, the presence of MMP14 was examined by immunohistochemistry (Fig. 5a, b; Supplementary Fig. 4). In uninjured vessels from wild-type and KLF6 heterozygous littermates, the presence of MMP14 was almost undetectable. By contrast, expression of MMP14 was clearly increased after injury compared with uninjured femoral arteries of wild-type mice. On vascular injury, MMP14 was mainly localized in ECs of the hyperplasic neointima and in the thicker tunica media, composed mainly by vascular smooth muscle cells (vSMCs) (Fig. 5a). Interestingly, MMP14 staining in injured vessels of *Klf6*^+/−^ mice was significantly decreased relative to control wild-type littermates (Fig. 5a, b). These results suggest that KLF6 regulates the expression of MMP14.

### MMP14 gene is a transcriptional target of KLF6

To assess whether MMP14 is a transcriptional target of KLF6, transient transfection experiments were performed in HEK293T cells (Fig. 5c, d). Ectopic expression of KLF6 led to a clear increase in the levels of endogenous MMP14 transcripts, as quantified by qRT-PCR (Fig. 5d). Also, the simultaneous overexpression of KLF6 and full length endoglin was associated with an increase in the levels of soluble endoglin (Fig. 5e). Furthermore, overexpression of KLF6 in HUVECs increased the levels of endogenous endoglin and MMP14 transcripts (Fig. 5f–h), as well as the amount of soluble endoglin under basal conditions and after endothelial denudation for 6 h (Fig. 5i). By contrast, suppression of KLF6 in HUVECs (Fig. 1d) decreased the levels of soluble endoglin induced during the wound healing process (Fig. 5j). These results suggest that the upregulated MMP14 proteolytically targets membrane endoglin releasing its soluble form to the culture media.

**Fig. 6:**
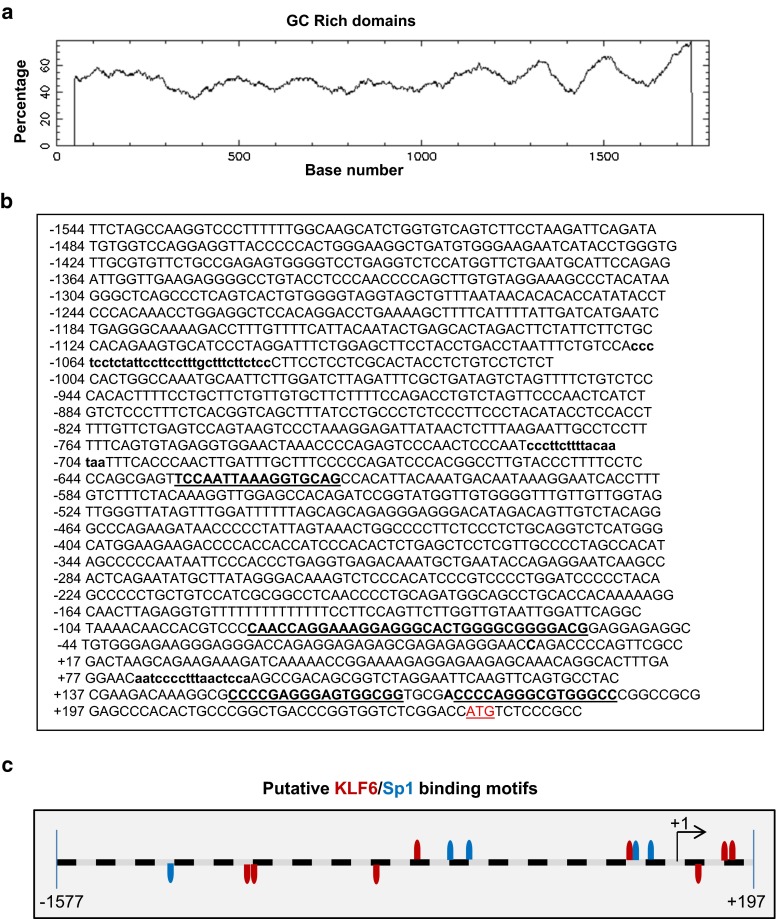
*In silico* analysis of putative KLF6 binding motifs in the MMP14 promoter. **a** GC-rich domains in the MMP14 promoter fragment −1544/+247. The percentage of GC bases along the promoter is shown. Several GC-boxes that recruit members of the Sp1/KLF transcription factors family were identified. **b** Putative transcription factor binding motifs for KLF6 (*bold letters*) in the plus (*capitals*) and negative (*lowercase letters*) strands of human MMP14 5′-proximal promoter sequence are indicated. KLF6(+); klf6(−). The translation initiation codon is marked in *red*. The numbering of the nucleotides is referred to the predicted transcription initiation site (+1) identified by the Genomatix MatInspector software. **c** Potential KLF6 (*red*) and Sp1 (*blue*) binding sites on the MMP14 gene promoter as identified by the Genomatix MatInspector software

**Fig. 7:**
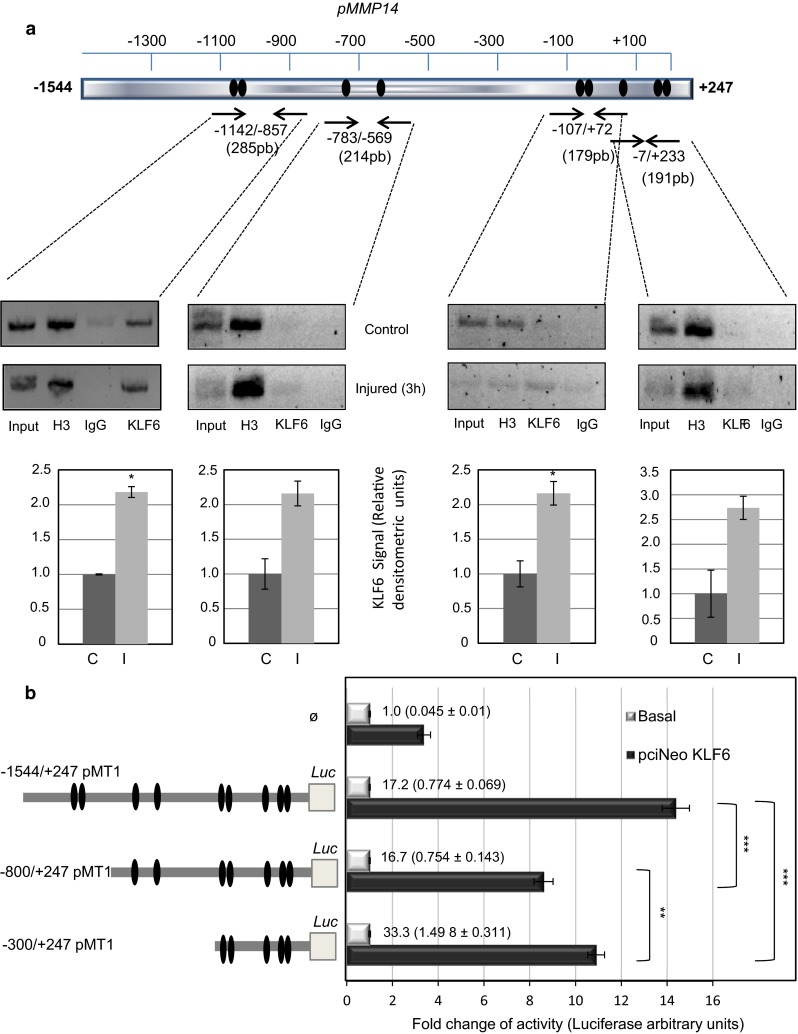
KLF6 interacts with MMP14 gene promoter and regulates its activity. **a** KLF6 interacts with MMP14 promoter in HUVECs. *In silico* analysis revealed the presence of potential KLF6-binding sites (*black ovals*) within GC-rich domains on MMP14 gene promoter using Genomatix MatInspector software (scheme in *upper panel*). Chromatin immunoprecipitation (ChIP) experiments of KLF6 over the 5′-proximal MMP14 promoter were carried out in HUVECs comparing control condition and after 3 h of wound healing (*middle panel*). The chromatin was digested obtaining a 150–300-bp fragments-enrichment. Immunoprecipitation was carried out with anti-KLF6, anti-H3K4 (positive control) and non-immune (IgG) antibodies. PCRs were done with primers to detect four regions containing putative KLF6-sites in the promoter region of MMP14, using the total lysate as an input of the sample. KLF6 binding to the different pMMP14 regions from the ChIP experiment was measured by densitometry of the individual bands, and values of the (KLF6-IgG)/Input ratios were represented (*bottom panel*). Normalized ratios in basal conditions (C) and 3 h after the endothelial injury (I) are shown. **b** KLF6 transactivates the MMP14 promoter. *Left* 5′-deleted construct series of the MMP14 promoter cloned into pGL2-luc. The size of each construct compared with the size of the whole promoter construct is shown in the scheme. The drawing is not to scale. *Right* HEK293T was co-transfected with a luciferase reporter driven by different MMP14 promoter constructs in pGL2-luc (−1544/+247 pMT1; −800/+247 pMT1; and −300/+247 pMT1) which include different putative binding GC-rich motifs for KLF6, and the expression vector pCIneo-KLF6. The pGL2-Luc empty vector (Ø) was used as a negative control. Overexpression of KLF6 upregulates between 8 and 14 times the activities of the MMP14 promoter constructs. The relative promoter activity under basal conditions is indicated in the corresponding bars, and the actual values of luciferase activity are shown in parenthesis. Transfection efficiency was corrected by relating luciferase activity to β-galactosidase activity. Results are expressed as a fold induction with respect to the basal activity of the corresponding constructs (**p < 0.01; ***p < 0.001)

To analyze whether the MMP14 gene promoter contains putative binding motifs for KLF6, an in silico analysis of the proximal 5′-MMP14 promoter region with Genomatix MatInspector software was carried out (Fig. 6). All proteins of the KLF family are characterized by a highly conserved DNA binding domain, which preferentially recognizes sequences designated as Sp1 sites, i.e., “GC-box” or “CACCC elements” of diverse target genes [32]. We found several GC-rich domains along the −1444/+235 fragment of the MMP14 promoter (Fig. 6), and consensus motifs for KLF6 were identified at positions −1067, −1048, −718, −635, −86, −65, +83, +152 and +175 (Fig. 6b, c). Next, the physical interaction between KLF6 and the putative KLF6 binding motifs on the MMP14 promoter was examined by chromatin immunoprecipitation. HUVEC monolayers were subjected to endothelial denudation, and chromatin immunoprecipitation experiments were assayed using an anti-KLF6 antibody, both in control situation and after 3 h of endothelial denudation. KLF6-immunoprecipitated chromatin was subjected to polymerase chain reaction using different couples of primers, encompassing the four clusters of KLF6 motifs present in the MMP14 promoter sequence. As shown in Fig. 7a, KLF6 binding to MMP14 promoter was detected in the four amplified fragments (−1142/−857, −783/−569, −107/+72 and −7/+233) under basal conditions. Moreover, on endothelial wounding, the binding of KLF6 to MMP14 promoter was enhanced, as shown in the corresponding densitometric analysis (Fig. 7a). These results indicate that at least one KLF6 motif within each cluster is bound to the MMP14 promoter. To study the functional effect of this interaction, transcriptional experiments using MMP14 promoter (pMT1) constructs were performed. Transient co-transfections in HEK293T cells with three different luciferase-pMT1 deletion constructs, all of them including putative binding sites for KLF6 (−1544/+247; −800/+247; and −300/+247), showed a marked activation (~8–14-fold) upon KLF6 overexpression (Fig. 7b). The highest stimulation (14-fold) was obtained with the longest construct (−1544/+247), which contains all the identified consensus KLF6 motifs. These results demonstrate that KLF6 is able to functionally interact with the proximal −1544/+247 fragment of the MMP14 promoter, stimulating its transcriptional activity.

## Discussion

An abnormal process of vascular remodeling may lead to pathological vascular lesions. Therefore, identification of molecular mediators implicated in the response to vascular injury is key to understand the mechanisms involved in vascular repair. Accumulated evidence in recent years implicates the transcription factor KLF6 in orchestrating the gene expression response of ECs to vascular injury [31, 33]. Endothelial expression of KLF6 is upregulated upon vascular injury and is responsible for the transcriptional activation of several genes involved in vascular remodeling, including endoglin, ALK1, urokinase plasminogen activator, collagen α1(I), TGF-β1 and TGF-β receptor type I [34, 35, 38]. Here, we provide evidence supporting the inclusion of MMP14 in the list of vascular genes regulated by KLF6. Using in vitro and in vivo models, we show that MMP14 is upregulated at the surface of ECs after vascular injury. Following endothelial wound healing, we found a temporal relationship between MMP14 and KLF6 transcript upregulation that is compatible with a transcriptional involvement of KLF6 in MMP14 gene expression regulation. Supporting this observation, MMP14 protein levels are much lower in injured femoral arteries of *Klf6*^+/−^ mice than those of wild-type animals. Also, suppression of KLF6 in cultured ECs decreases MMP14 expression and the subsequent release of soluble endoglin. Furthermore, ectopic expression of KLF6 is able to markedly transactivate the MMP14 gene promoter construct specifically designed and generated for this purpose. Previous reports have postulated that E2F and Ets families of transcription factors play a role in cancer through regulation of MMP14 gene promoter activity [43, 44]. In addition, transcription factors Egr-1, Sp1 and Sp3 are involved in the regulated expression of MMP14 in endothelial cells driven by hemodynamic forces and extracellular matrix contacts [45–47]. To our knowledge, this is the first study that analyzes the transcriptional activity of an MMP14 gene promoter construct in response to endothelial injury. Our results prompted us to explore the potential regulation by KLF6 of the MMP14 gene. An in silico analysis of the proximal MMP14 promoter sequence revealed the presence of nine putative consensus binding sites for KLF6 along the −1544/+247 pMT1 fragment. The motifs are located at positions −1067, −1048, −718, −635, −86, −65, +83, +152 and +175. By chromatin immunoprecipitation, we demonstrate not only that KLF6 binds to four different KLF6 clusters (−1142/−857, −783/−569, −107/+72 and −7/+233) of the MMP14 promoter, but we also show that this binding is increased on endothelial injury in vitro. This finding agrees with the functional effect of KLF6 in transcriptional experiments using several MMP14 promoter constructs. Indeed, all constructs, including putative binding sites for KLF6 (−1544/+247; −800/+247; and −300/+247), showed a marked activation upon ectopic expression of KLF6. Together, these results demonstrate that KLF6 is able to functionally interact with the MMP14 promoter, stimulating its transcriptional activity. Importantly, many of the KLF6 motifs in the proximal MMP14 promoter are located at GC-rich regions where they overlap, or are surrounded by, consensus binding sites for the transcription factor Sp1 (Fig. 6). The physical proximity between KLF6 and Sp1 motifs suggests a synergistic collaboration of both transcription factors in the transcriptional machinery complex of MMP14. Supporting this interpretation, we have previously demonstrated the direct physical interaction and functional cooperation between Sp1 and KLF6 on endoglin and ALK1 gene promoters, in response to vascular injury [34, 35, 48].

Here, we report for the first time that endothelial denudation in vitro leads to an increase in soluble endoglin levels. Interestingly, MMP14 co-localizes with and targets membrane-bound endoglin to proteolytically release soluble endoglin. Overall, the functional link between MMP14 and endoglin in their parallel gene expression in response to endothelial damage supports their coordinated involvement in the molecular events triggered by a vascular injury to recover the endothelial homeostasis. While the role of MMP14 in vascular remodeling and the cellular processes involved has been previously described [17, 49–51], little is known about the role of soluble endoglin in this specific setting. Circulating levels of soluble endoglin are elevated in preeclampsia, hypercholesterolemia, diabetes, atherosclerosis, severe pulmonary arterial hypertension, acute myocardial infarction and some types of solid cancers [27–29, 52–54]. Of note, many of these pathologies are associated with cardiovascular damage. At variance with the pro-angiogenic role of membrane-bound endoglin, soluble endoglin displays an anti-angiogenic activity [25, 27, 30], which has been postulated as a pathogenic contributor in preeclampsia [27]. In the context of cardiovascular repair after injury, active angiogenesis and endothelial cell proliferation are expected to occur in order to reestablish the proper circulation in the tissues. KLF6, an upstream trigger of soluble endoglin, is strongly expressed in ECs of highly vascularized tissues such as placenta and lung, and promotes vascular remodeling [31]. Thus, it is intriguing the role of the anti-angiogenic activity associated with soluble endoglin during the vascular repair process. Probably, the release of soluble endoglin constitutes an early response to vascular damage that halts angiogenesis, followed by a second phase involving the upregulation of membrane-bound endoglin and active angiogenesis [6, 35, 55]. In this regard, the release of soluble endoglin may have important consequences at the cellular level as it is associated with the decrease in the surface endoglin, which may lead to the downregulation of the ALK1/endoglin-dependent signaling pathway, a route that in ECs promotes angiogenesis [6, 36]. Further studies are necessary to elucidate the exact role of soluble endoglin, and the associated downregulation of membrane-bound endoglin, on the vascular repair process.

Although we have focused our MMP14 studies on its endothelial expression and function, MMP14 can be expressed by other cell types including vSMCs [49, 50]. This is in agreement with the expression of MMP14 in the layer of vSMCs surrounding the tunica intima after endothelial denudation of the femoral artery as seen in Fig. 5a. Of note, vSMCs can also express the MMP14 substrate endoglin in pathological vessels [56]. At variance with ECs, vSMCs are not directly injured in this animal model. Thus, a cross-talk between endothelial and smooth muscle cell layers appears necessary to explain the upregulated expression of MMP14 in vSMCs on vascular injury. In this line, an increase in IL-6 along the in vitro wounding process of ECs has been reported [34]. Moreover, KLF6 regulates the transcriptional activity of IL6 gene, suggesting that IL6 is a putative candidate to contribute to the paracrine effect on vSMCs surrounding the endothelium [34]. Supporting the involvement of IL6 in the vascular repair process is the finding that IL-6 KO mice take three times longer to heal than wild-type animals [57]. Thus, it can be postulated that the release of soluble factors, such as IL-6, from the injured endothelium would serve to expand the repair signal from the inner to the more distant layers of the vessel. This coordinated cross-talk between vSMCs and ECs [50] and the upregulated expression of MMP14 in vSMCs upon vascular damage are in agreement with the critical role that vSMCs play during pathologic vascular remodeling. Thus, VSMCs embedded within the collagen-rich matrix of the artery wall mobilize and activate MMP14 to degrade and infiltrate 3D barriers of interstitial collagen, including the arterial wall. In fact, genetic deletion of MMP14 affords mice with a protected status against neointimal hyperplasia and lumen narrowing in vivo [49].

**Fig. 8:**
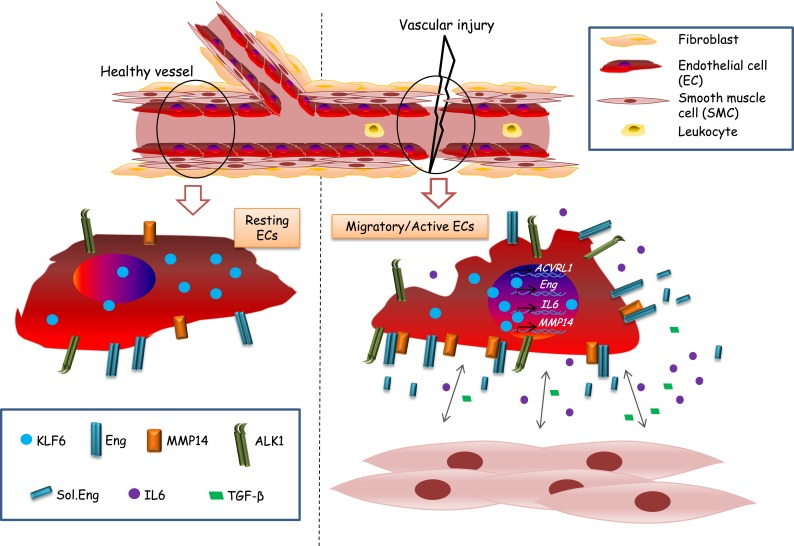
Hypothetical model of KLF6 regulation during vascular injury. Upon vascular injury, KLF6 translocates from the cytoplasm to the nucleus and activates endothelial genes such as endoglin [35], ALK1 [34], IL6 [34] and MMP14 (this report). Endoglin and MMP14 co-localize at the cell surface. Then, MMP14 triggers the release of soluble endoglin. Soluble proteins such as IL6, endoglin and TGF-β are potential players in the cross-talk between ECs and vSMCs during wound healing

In summary, our studies demonstrate for the first time a functional relationship between the transcription factor KLF6 and MMP14 expression (Fig. 8). Also, after vascular injury, KLF6 is responsible for the increased expression of membrane endoglin in endothelial cells and the subsequent MMP14-dependent release of soluble endoglin. The levels of IL6, a proangiogenic and proinflamatory cytokine, are also increased, as a consequence of KLF6 activation. Both IL6 and soluble endoglin could contribute to the cross-talk between ECs and vSMCs, which is involved in the mechanism of wound healing. Overall, these results suggest that KLF6 is a master regulator during vascular injury, repair and migration, and can play a role in physiological and pathological processes of the blood vessels.

## Electronic supplementary material

Below is the link to the electronic supplementary material.

Supplementary material 1 (PDF 1321 kb)
