# Supplementary material for: Transcription factor KLF6 upregulates expression of metalloprotease MMP14 and subsequent release of soluble endoglin during vascular injury
Source: Angiogenesis. 2016 Feb 5;19:155–71. doi: 10.1007/s10456-016-9495-8 (PMC4819519; doi:10.1007/s10456-016-9495-8)
Supplement: Supplementary file 1 — Supplementary material 1 (PDF 1321 kb) [file 10456_2016_9495_MOESM1_ESM.pdf]

# SUPPLEMENTARY MATERIAL

TRANSCRIPTION FACTOR KLF6 UPREGULATES EXPRESSION OF METALLOPROTEASE MMP14 AND SUBSEQUENT RELEASE OF SOLUBLE ENDOGLIN DURING VASCULAR INJURY

Eunate Gallardo-Vara[1], Francisco J. Blanco[1], Mercè Roqué[2], Scott L. Friedman[3], Toru Suzuki[4], Luisa M. Botella[1], Carmelo Bernabeu[1*]

[1]Centro de Investigaciones Biológicas, CSIC, and Centro de Investigación Biomédica en Red de Enfermedades Raras (CIBERER), 28040 Madrid, Spain

[2]Servei de Cardiologia, Hospital Clínic i Institut d'Investigacions Biomèdiques August Pi i Sunyer, Barcelona, Spain

[3]Division of Liver Diseases, Icahn School of Medicine at Mount Sinai, New York, NY, USA

[4]Department of Cardiovascular Sciences, University of Leicester, Leicester, UK, National Institute for Health Research Leicester Cardiovascular Biomedical Research Unit, Glenfield Hospital, Leicester, UK

*Corresponding author: Carmelo Bernabeu, Centro de Investigaciones Biologicas, c/ Ramiro de Maeztu 9, Madrid 28040, Spain; Phone: 34-918373112 x 4246; E-mail: bernabeu.c@cib.csic.es

**Supplementary Fig. 1. Silencing of MMP14 expression in HUVECs decreases cell migration and soluble endoglin release during wound healing.** HUVECs were nucleofected with a mix of three different siRNA-MMP14 [NM_004995, Sigma] (siRNA-MT1) or Scrambled siRNA [Universal negative control SIC001, Sigma] (Sc), the nucleofection efficiency being ~40%. After nucleofection, the levels of MMP14 transcripts were measured by quantitative RT-PCR (**a**). Monolayers of nucleofected HUVECs were wounded in vitro, leaving approximately 40% intact of total monolayer. Photographs (5X) were taken at different times after wounding (**b**) and the percentage of wound closure (**c,d**) and the levels of soluble endoglin (**e,f**) were measured at 6h and 8h. Suppression of MMP14 significantly decreases the migration phenotype and the release of soluble endoglin (***$p<0.001$ respect to scrambled siRNA condition).

**Supplementary Fig. 2. Effect of KLF6 suppression on MMP14 and endoglin colocalization.** KLF6 suppression was carried out by nucleofection in HUVECs with siRNA specific for KLF6 (siKLF6), using scrambled siRNA as a control. After forty eight hours, HUVEC monolayers were wounded in vitro and the presence of MMP14 and endoglin was detected by immunofluorescence at the indicated time points. Cells were incubated with a mouse antibody anti-endoglin (P4A4) or a rabbit antibody anti-MMP14, followed by a secondary anti-mouse IgG coupled to Alexa 488 (green staining) or a secondary anti-rabbit IgG coupled to Alexa 647 (red staining). (**a**) Single stainings and merge images plus DAPI (nuclear staining in blue) are shown at the indicated magnification (63x). Colocalization of MMP14 and endoglin is visualized by the yellow color. **(b-d)** Quantification studies. **(b)** Representative merge images showing the colocalization of MMP14 and endoglin with white dots (first row) and the corresponding cytofluorograms (second row). **(c)** Quantification of MMP14 and endoglin colocalization (percentage) obtained from the cytofluorograms. **(d)** Quantification of endoglin and MMP14 expression relative to nuclei staining with DAPI. This is a representative experiment of 3 different ones.

**Supplementary Fig. 3. Coimmunoprecipitation of endoglin and MMP14.** HUVECs were wounded *in vitro* and lysed at times 0h and 6h. When necessary, HUVECs were nucleofected 48 hours before wounding. HUVEC were lysed with lysis solution (1% NP40, 150 mM NaCl, 50 mM Tris, 0.5 mM NaF, 1 mM PMSF and protease and phosphatase inhibitors). Total cell lysates were subjected to immunoprecipitation (IP) with anti-MMP14 monoclonal antibody (1:80 dilution, #ab51074, Abcam) coupled to Protein G magnetic beads (Millipore). Immunoprecipitates were separated by SDS-PAGE on 8% acrylamide gels under reducing conditions and electrotransferred to a nitrocellulose membrane. Western Blot (WB) analysis was carried out using the P4A4 mouse monoclonal antibody to endoglin and a secondary antibody conjugated to Horse Radish Peroxidase (HRP). The membranes were developed with Supersignal west Pico-chemiluminescent substrate (Pierce) to enhance HRP luminescence, following the manufacturer´s instructions. Quantification of the bands intensity was measured by Quantity one software (Bio-Rad, Chicago, IL). A negative control (NC) with an IgG matched antibody (instead of anti-MMP14) is included. The molecular weight markers (M) are in the last lane on the right. Representative blots are shown on the left, whereas the normalized intensity of the endoglin bands is shown in the right histograms. (**a**) HUVECs were nucleofected with the KLF6 expression vector pciNeoKLF6 or the corresponding empty vector, as indicated. (**b**) HUVECs were nucleofected with the siRNA specific for KLF6 (siKLF6) or an scrambled siRNA (siScrambled), as indicated.

**Supplementary Fig. 4. Negative control for immunohistochemical staining of MMP14 in mouse femoral arteries.** Tissue samples were processed for immunochemistry as in Fig. 5a, but in the absence of primary antibody. Pictures were taken at ×50 magnification.

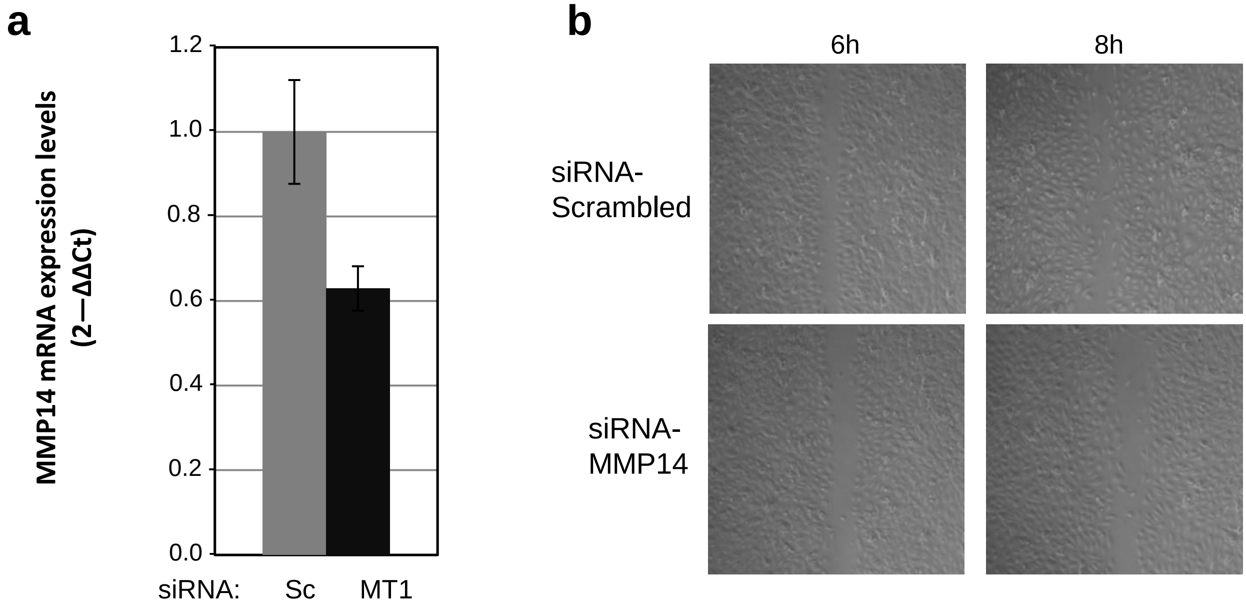


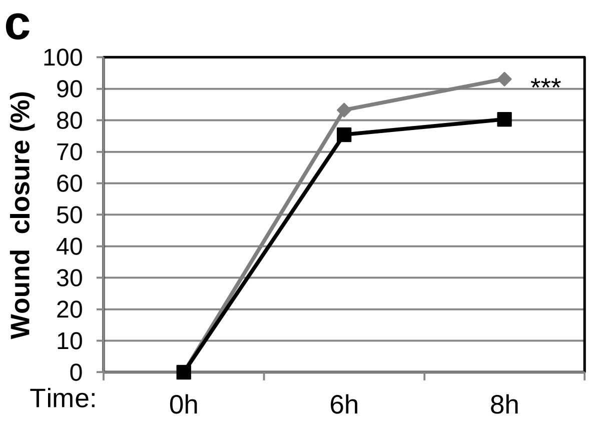


d

| | % Wound closure | |
|---|---|---|
| | **6h post-wounded** | **8h post wounded** |
| **siRNA-Scrambled** | 83.24 ± 1.63 | 93.08 ± 4.82 |
| **siRNA-MT1** | 75.42 ± 4.37 | 80.30 ± 5.69 |
| **p-value** | 0.087054181 | 0.000152661 |

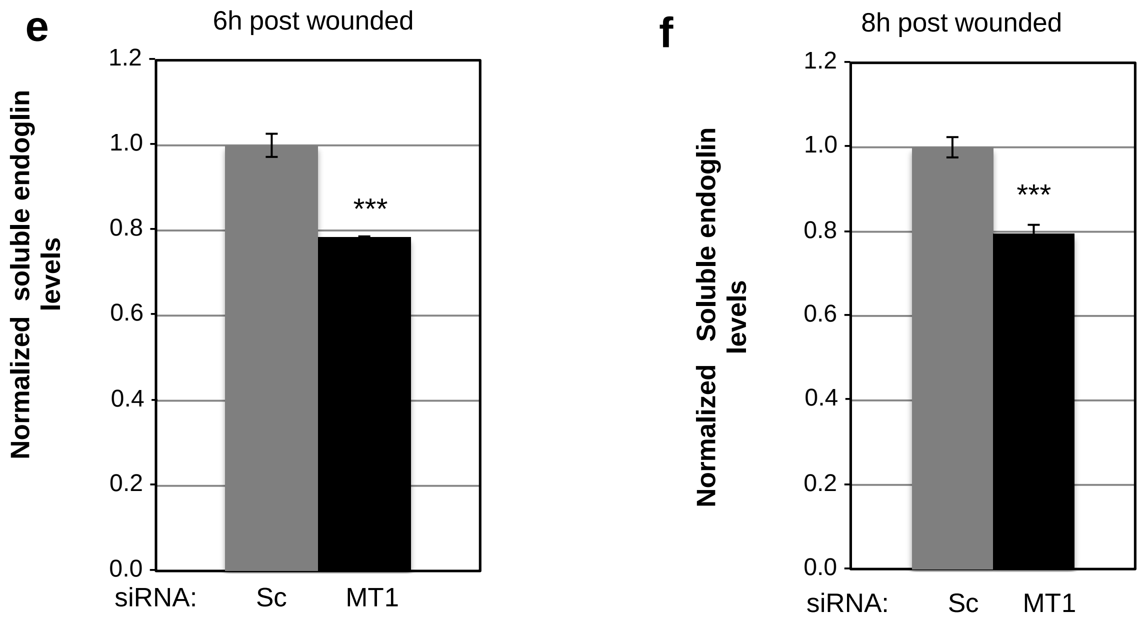


Supplementary Figure 1

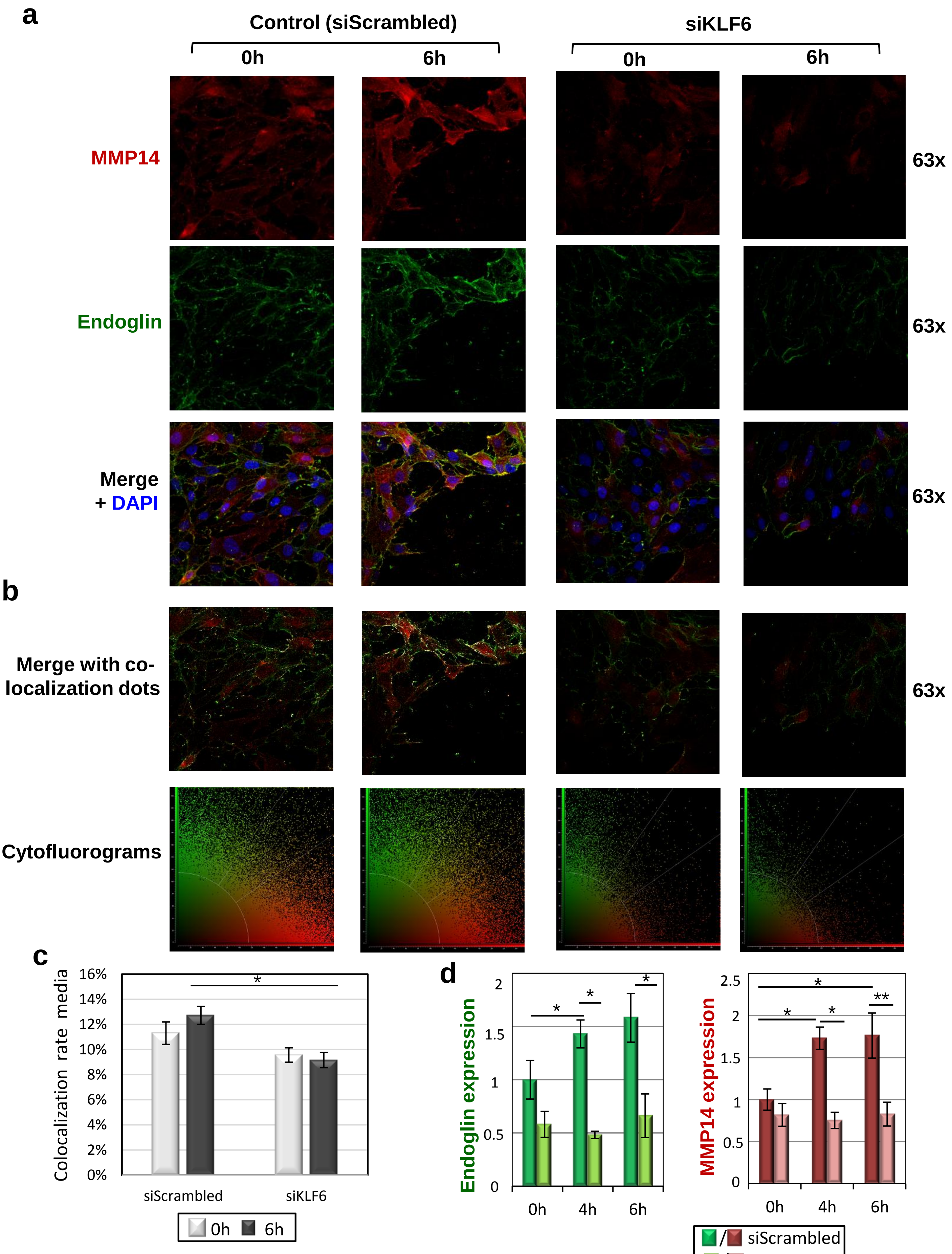


Supplementary Figure 2

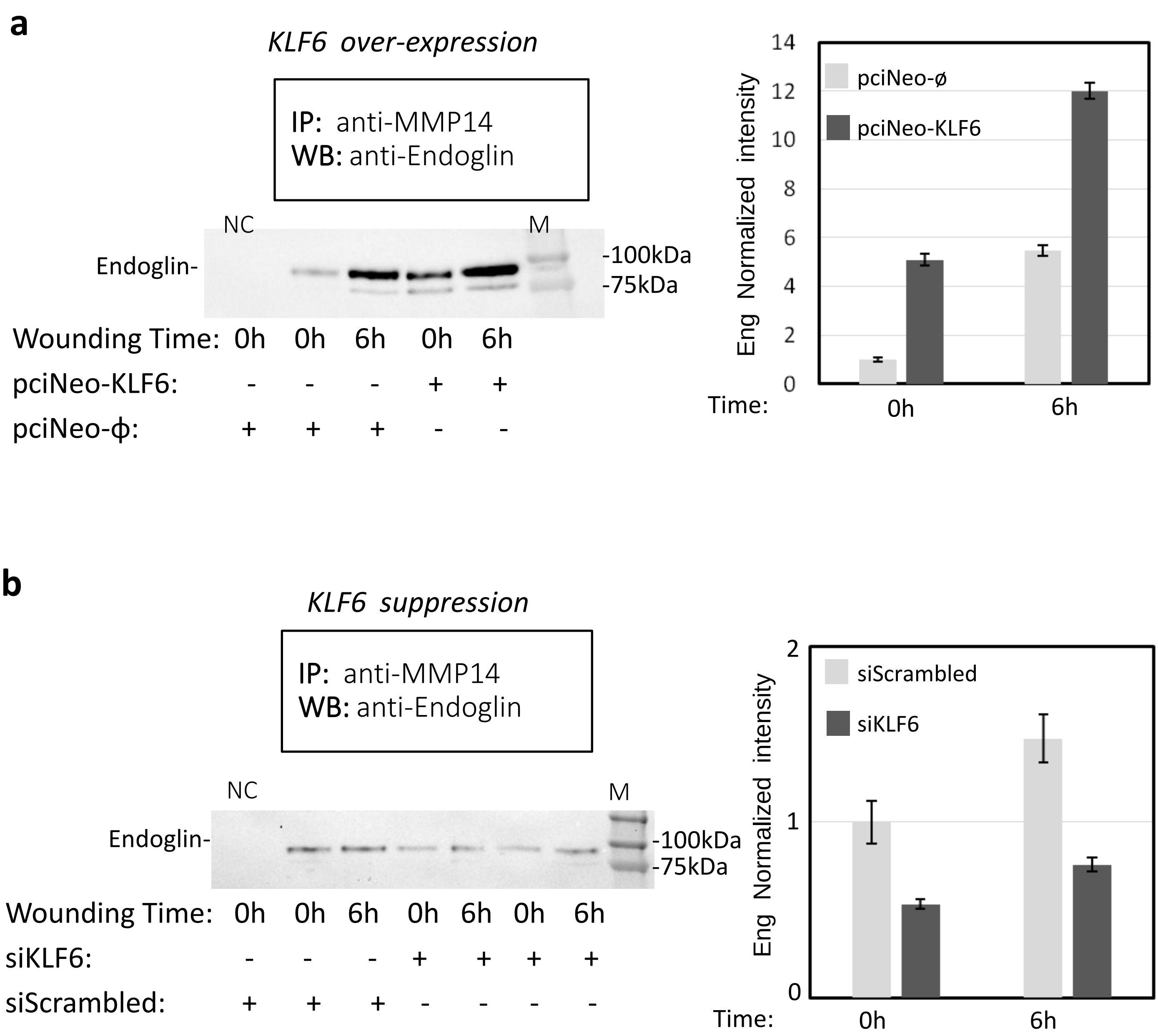


Supplementary Figure 3

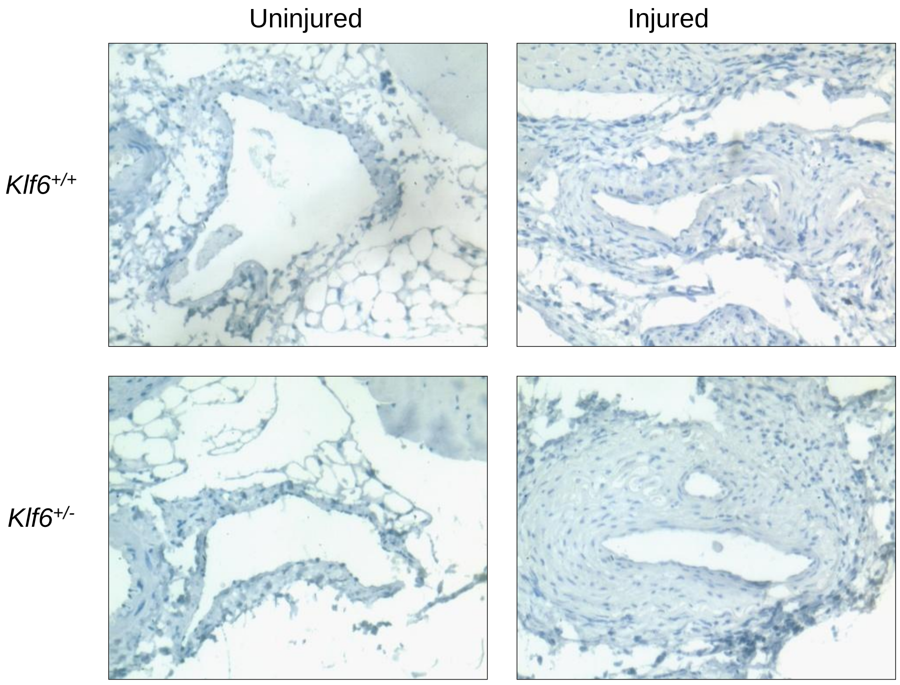


Supplementary Figure 4
